# Processing Phage Therapy Requests in a Brussels Military Hospital: Lessons Identified

**DOI:** 10.3390/v11030265

**Published:** 2019-03-17

**Authors:** Sarah Djebara, Christiane Maussen, Daniel De Vos, Maya Merabishvili, Benjamin Damanet, Kim Win Pang, Peggy De Leenheer, Isabella Strachinaru, Patrick Soentjens, Jean-Paul Pirnay

**Affiliations:** 1 Center for Infectious diseases ID4C, Queen Astrid military hospital, Bruynstraat 1, B-1120 Brussels, Belgium; christiane.maussen@mil.be (C.M.); benjamin.damanet@mil.be (B.D.); winggopang@gmail.com (K.W.P.); peggy.deleenheer@mil.be (P.D.L.); isabella.strachinaru@mil.be (I.S.); Patrick.Soentjens@mil.be (P.S.); 2 Laboratory for molecular and cellular technology, Queen Astrid military hospital, Bruynstraat 1, B-1120 Brussels, Belgium; danielmarie.devos@mil.be (D.D.V.); maia.merabishvili@mil.be (M.M.); jean-paul.pirnay@mil.be (J.-P.P.)

**Keywords:** bacteriophages, phage therapy, antibiotic resistance, *Pseudomonas aeruginosa*, *Escherichia coli*, *Staphylococcus aureus*, Brussels, Belgium

## Abstract

There is a growing interest in phage therapy as a complementary tool against antimicrobial resistant infections. Since 2007, phages have been used sporadically to treat bacterial infections in well-defined cases in the Queen Astrid military hospital (QAMH) in Brussels, Belgium. In the last two years, external requests for phage therapy have increased significantly. From April 2013 to April 2018, 260 phage therapy requests were addressed to the QAMH. Of these 260 requests, only 15 patients received phage therapy. In this paper, we analyze the phage therapy requests and outcomes in order to improve upon the overall capacity for phage therapy at the QAMH.

## 1. Introduction

Antibiotic resistance is an increasing threat not only to human health but also to the production of food and to sustainable development. By 2050, it is estimated that antimicrobial resistant infections will kill more than 10 million people per year (more than cancer), and the cost in terms of lost global production will amount to 100 trillion USD, if no action is undertaken [1]. In 2016, the United Nations acknowledged that the current antimicrobial resistance crisis is mainly due to the inappropriate use of antimicrobial medicines in the public health, animal, food, agriculture, and aquaculture sectors; a lack of access to health services (including to diagnostics and laboratory capacity); and the presence of antimicrobial residues in soil, crops, and water. They subsequently committed to work at national, regional, and global levels to support the development of new antimicrobial agents and therapies [2]. In 2017, the World Health Organization published a list of 12 drug-resistant bacteria for which new antibiotics are urgently needed. The critical priority category consisted of *Acinetobacter baumannii* (carbapenem-resistant), *Pseudomonas aeruginosa* (carbapenem-resistant), and *Enterobacteriaceae* (carbapenem-resistant, ESBL-producing) [3]. In the US, Rice coined the term “ESKAPE” pathogens (*Enterococcus faecium*, *Staphylococcus aureus, Klebsiella pneumoniae, Acinetobacter baumannii, Pseudomonas aeruginosa*, and *Enterobacter* spp.) to emphasize that these bacteria currently cause the majority of US hospital infections and effectively “escape” the effects of antibacterial drugs [4]. However, only a few new antibiotics are being developed, and none are expected to be effective against the most dangerous antibiotic-resistant bacteria called “superbugs” [5]. There is renewed interest in phage therapy as an alternative or addition to antibiotic therapy for the treatment of bacterial infections. Bacteriophages (phages for short) are viruses that target and infect a subset of bacteria with almost no collateral damage to the commensal flora (e.g., the gut and skin microbiomes). Phage therapy was first introduced in Western medicine in the 1920s. Upon the widespread marketing of antibiotics, which had the advantage of exhibiting a broad spectrum antimicrobial activity, phage therapy was abandoned in the West by about the 1940s. It continued to be developed and used in Eastern Europe and in former Soviet Republics, with the Eliava Institute in Tbilisi (Georgia) as the epicenter [6]. In 2007, the Queen Astrid military hospital (QAMH) in Brussels was the first Belgian hospital to reinitiate a focus on phage therapy, and this under the umbrella of article §37 (unproven interventions in clinical practice) of the Declaration of Helsinki, which was developed by the World Medical Association [7].

*Article §37. In the treatment of an individual patient, where proven interventions do not exist or other known interventions have been ineffective, the physician, after seeking expert advice, with informed consent from the patient or a legally authorised representative, may use an unproven intervention if, in the physician’s judgement, it offers hope of saving life, re-establishing health, or alleviating suffering. This intervention should subsequently be made the object of research, designed to evaluate its safety and efficacy. In all cases, new information must be recorded and, where appropriate, made publicly available.*

**Figure 1:**
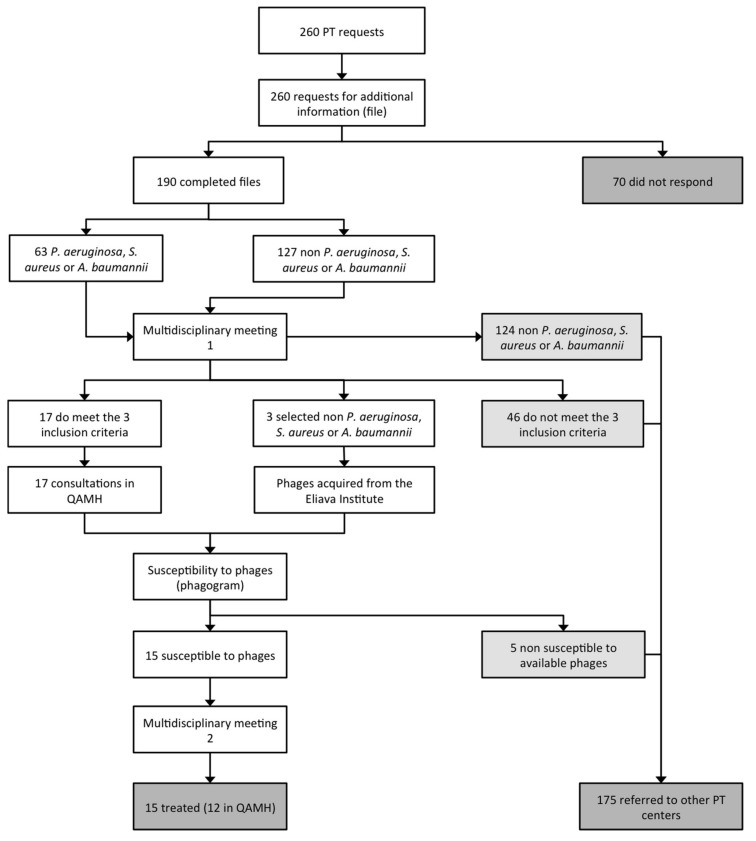
Patient care workflow in phage therapy at the Queen Astrid military hospital in Brussels (Belgium). PT, phage therapy; QAMH, Queen Astrid military hospital.

Since then, patients have been occasionally treated by phage therapy at the QAMH. Last year, we reported the case of a patient treated with intravenous bacteriophage monotherapy (no antibiotics were used) against colistin-only-sensitive *P. aeruginosa* [8]. Belgium is now implementing a pragmatic phage therapy framework that centers on the magistral preparation (compounding pharmacies in the US) of tailor-made phage medicines [9], which can pave the way for a broader and more structured application of phages in Belgium. Most requests for phage therapy used to originate from within the QAMH, and more specifically from the burn wound center. Since 2017, however, a spectacular increase in external phage therapy requests to the QAMH has been observed, most of them related to the broadcast of two phage therapy prime time documentaries on Dutch television: *Bacteriofagen: een alternatief voor antibiotica?* (Bacteriophages: an alternative to antibiotics?) on the 21st of March 2017 [10] and *Dokters van Morgen over bacteriën* (Doctors of Tomorrow on bacteria) on the 24th of October 2017 [11]. One hundred and fifty-one phage therapy requests were registered in 2017, with increases in requests following the documentaries’ broadcast dates. A third Dutch documentary aired on 5 February 2019 [12] was again followed by a considerable increase in phage therapy request. Between April 2013 and April 2018, 260 phage therapy requests were addressed to QAMH medical staff by e-mail, post, or telephone. All these requests were re-directed to a centralized e-mail address (pt@mil.be), upon the receipt of which a reply was sent to request standardized medical information (Supplementary File 1) with regard to the particular case. One hundred and ninety applicants (73.1%) responded (Figure 1). The received medical files were stored in a dedicated access database to ensure a uniform follow-up.

The aim of this paper was to describe, analyze, and discuss the 260 phage therapy requests addressed to the QAMH to raise awareness for the increased interest in phage therapy in Northwest Europe and to guide future phage therapy R&D in and outside the QAMH.

## 2. Demographics

**Figure 2:**
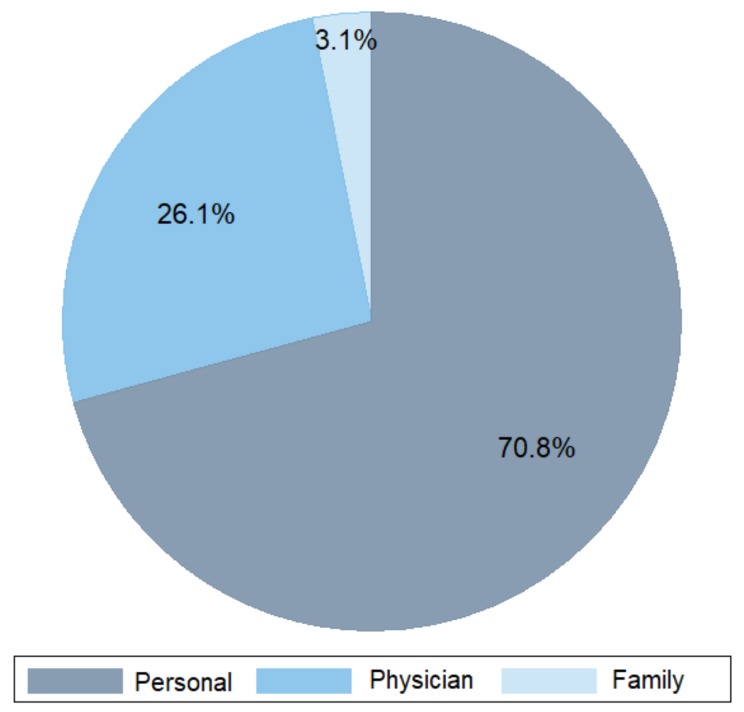
Initiators of the 260 phage therapy requests.

Most phage therapy requests were initiated by the patients themselves (70.8%), followed by physicians (26.1%) and the patient’s family (3.1%) (Figure 2). The increased attention for phage therapy in the popular media seems to have raised the awareness of patients to this new therapeutic alternative. Not surprisingly, the majority of phage therapy requests to the QAMH in Brussels (Belgium) originated from The Netherlands (66.9%), one of Belgium’s neighboring countries. The other countries of patients’ origins were, in decreasing order, Belgium (19.2%), France (7.3%), Germany (2.3%), and Luxembourg (1.1%) (Table 1). Fifty-three percent of the patients requesting phage therapy were male, and the mean age (SD) of the 132 patients, who communicated their age, was 57.9 (20.7) years (Table 1). Patients older than 60 years were more prevalent (61.4%) (Table 1).

## 3. Infection Types and Bacterial Pathogens

The infection types and their causative bacterial pathogens are shown in Table 1. Urinary tract infection (UTI) was the most common type of infection (31.8% of all requests), with chronic bladder infection as the most frequent UTI type. Less frequent were responders with neurogenic bladder and recurrent UTIs. The leading causative infectious agents in UTI were (in descending order) *Escherichia coli, Enterococcus faecalis, K. pneumoniae*, and *Enterobacter cloacae*.

**Figure 3:**
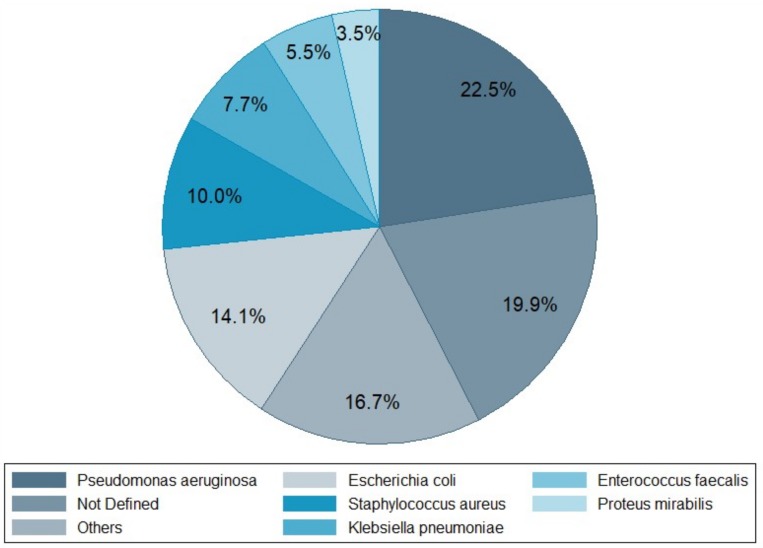
Relative prevalence of 311 reported bacterial pathogens (Table 1).

**Figure 4:**
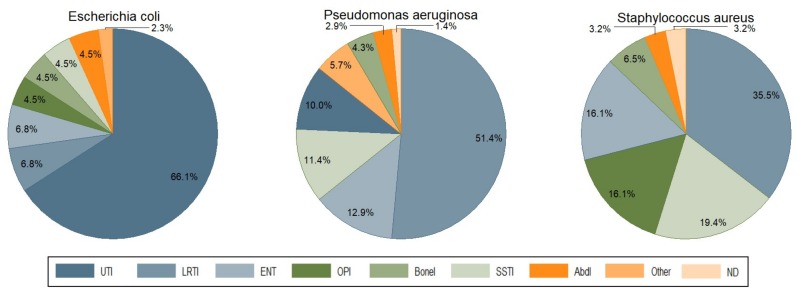
The proportion of the three most prevalent bacteria in the different infection types. AbdI, abdominal infection; BoneI, bone infection; ENT, ear-nose-throat; LRTI, lower respiratory tract infection; OPI, Orthopedic prosthesis infection; SSTI, skin and soft tissue infection; UTI, urinary tract infection.

**Figure 5:**
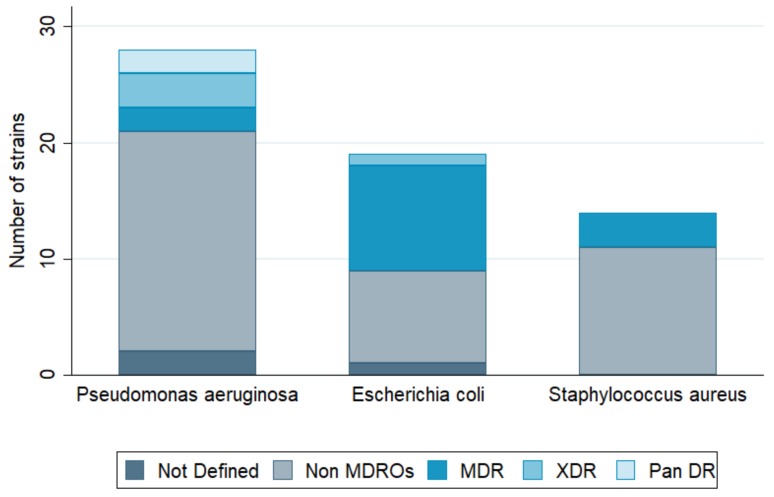
Proportion of drug-resistant strains in the three most prevalent bacterial pathogens (see also Supplementary Table S1). MDR, multidrug-resistant; non-MDROs, non-multidrug-resistant organisms; Pan DR, pandrug-resistant; XDR, extensively drug-resistant.

Lower respiratory tract infection (LRTI) was the second most frequent type of infection reported to request phage therapy (23.8%), with *P. aeruginosa* as the predominant respiratory pathogen. In this category, cystic fibrosis, bronchiectasis, chronic obstructive pulmonary disease (COPD), and asthma were the most common underlying pathologies. Third in line were bone infections (Bone Is) and orthopedic prosthesis infections (OPIs) with 12.1% of requests, including osteomyelitis, osteitis in diabetic foot, infected traumatic fractures, and hip and knee prosthesis infections. Ear, nose, and throat infections (ENTIs) came in fourth position (8.9%), with chronic sinusitis and chronic otitis as the main pathologies. Skin and soft tissue infections (SSTIs) were fifth, with 8.5% of requests. Burn and chronic wound infections (including postoperative surgical wounds and diabetic foot ulcers) were the most common SSTIs. Surprisingly, only few phage therapy requests concerned patients with abdominal infections (AbdIs) (3.6%). There were also 11 requests from patients seeking phage therapy for non-bacterial or non-infectious medical conditions such as arthritis, interstitial cystitis, cirrhosis, collage colitis, and irritable bowel disease. No fewer than 30 bacterial species were at the basis of the reported infections, with *P. aeruginosa* (22.5%) as the leading causative agent (Figure 3). This pathogen was found mostly in LRTIs (51.4%), and to a lesser extent in ENTIs, SSTIs, and UTIs (Figure 4). The second most prevalent bacterium was *E. coli* (14.1%), found mostly in UTI patients (66.1%) (Figure 4). The third one was *S. aureus* (10%), mostly found in LRTIs, ENTIs, OPIs, and SSTIs. Other frequently encountered bacteria were *Enterobacteriaceae*, including *K. pneumoniae* (7.7%, mainly in UTI), *E. faecalis* (5.5%), and *Proteus mirabilis* (3.5%) (Figure 3). Interestingly, *E. faecium*, which is often considered as the leading cause of multi-drug resistant enterococcal infections (over *E. faecalis*), was not represented. From the 190 requests with completed files, only 102 antibiograms could be retrieved and analyzed (Supplementary Table S1). Bacterial strains were classified in five different categories of acquired antibiotic resistance according to Magiorakos’ classification proposal [13]. Multidrug-resistant (MDR) was defined as non-susceptible to at least one agent in three or more antimicrobial categories, which Magiorakos and colleagues had previously constructed, for each of the organisms with the intent of placing antimicrobial agents into more therapeutically relevant groups. Extensively drug-resistant (XDR) was defined as non-susceptible to at least one agent in all but two or fewer antimicrobial categories (i.e., bacterial isolates remain susceptible to only one or two categories), and pandrug-resistant (PDR) was defined as non-susceptible to all agents in all antimicrobial categories (i.e., no agents tested as susceptible for that organism). Non-defined (ND) was used when the information given by the antibiogram (the result of an antibiotic susceptibility test) was incomplete to classify the germ into one of the five antibiotic resistance categories. We chose to focus on the three most encountered pathogens in our cohort (*P. aeruginosa*, *E. coli,* and *S. aureus*), for which, respectively, 28, 19, and 14 antibiograms were collected and analyzed (Figure 5). For *P. aeruginosa*, 7.1% of strains were classified as MDR, 10.7% as XDR, and 7.1% as PDR. The proportion of MDR *E. coli* strains was no less than 47.3% with 5.2% of XDR, but no PDR strains were observed. Approximately a fifth (21.4%) of *S. aureus* strains were MDR, and none were XDR or PDR. Notwithstanding the fact that these statistics are more or less in line with the literature with regard to the current antibacterial resistance crisis, we observed that—with the exception of *E. coli*—the majority of phage therapy requests concerned non-MDR organisms. Technically speaking, under the umbrella of article 37 of the Declaration of Helsinki, phage therapy can only be applied when proven (e.g., antibiotic) therapies are ineffective. So, when there are indications (e.g., based on an antibiogram) that the infection can be treated with an antibiotic, phage therapy should not be considered.

## 4. Patient Care Workflow

A dedicated patient care workflow in phage therapy was created to ensure an accurate and systematic monitoring of phage therapy requests, treatments, and follow-up (Figure 1).

When the medical dossier was complete, which was the case for 190 responders; the case was discussed by dedicated infectious disease specialists and microbiologists during a first multidisciplinary meeting. Three inclusion criteria were taken into account:

- Infection with *S. aureus*, *P. aeruginosa*, and/or *A. baumannii*, the three bacterial pathogens against which the QAMH possessed potent phages [14,15];
- Bacterial infection associated with antibiotic treatment failure;
- The absence of other therapeutic options.

When eligibility criteria were met, which was the case for only 20 patients (Figure 1), a consultation with an infectious disease specialist was scheduled, during which a physical examination and an anamnesis of the patient were performed and bacterial samples of the infection site(s) were taken. Consecutively, these bacterial samples were sent to the clinical laboratory for standard bacterial culture, isolation, and identification (using VITEK II, bioMérieux, Marcy l’Etoile, France). For *P. aeruginosa*, *S. aureus*, and *A. baumannii* isolates, a “phagogram” (by analogy with an antibiogram) was performed, based on the spot-test and the double-agar overlay method (both methods are described in Kakabadze et al. [16]), to determine their susceptibility to the phages available (for non-commercial R&D purposes) in the QAMH and described in Merabishvili et al. [14,15]. For the spot test, 100 µL of bacterial suspension at a concentration of 10^8^–10^9^ cfu/mL was mixed with 3.5 mL of LB (Lysogeny Broth, Becton Dickinson, Franklin Lakes, NJ, USA) medium with 0.6% agar (Becton Dickinson) at 45 °C and poured on petri plates containing a solidified bottom layer of LB medium with 1.5% agar. After air-drying, 10 μL of serial 100-fold dilutions of phage cocktails were spotted on the bacterial lawn. Plates were incubated overnight at 37 °C. Clearance zones (when present) were examined the next day. In case phage activity was observed against the tested bacterial strain, the double-agar overlay method was applied to determine the Efficiency of Plating (EOP) of the phage cocktails, calculated as the ratio of activity in the test strain (i.e., the patient’s strain) to the activity on the host strain (i.e., the production strain). In 15 cases, the phagogram indicated susceptibility of the infecting bacterial pathogen(s) to the tested phage(s) and treatment was proposed to the patient and to his treating physician (Figure 1). Treatment protocols and patient follow up were discussed during a second multidisciplinary meeting. Details with regard to the phage therapy protocol and outcome will be the subject of separate publications (grouped according to medical indications and authored by the different treating physicians) and will not be discussed in this article. However, we can disclose that no serious adverse events were observed and that, in general, phage therapy seemed helpful in a considerable number of the cases. We also would like to stress that, with the exception of one case that was recently published [8], other antimicrobial agents (e.g., antibiotics) were applied simultaneously with the phages.

## 5. Implications for Future Activities

Most phage therapy requests were initiated by the patients themselves, which in part could explain the low proportion of MDR infections and the occurrence of requests for phage therapy against non-bacterial infections. The role of the media was non-negligible in the patients’ self-management of their disease, as demands increased spectacularly immediately after two prime-time TV-shows promoting phage therapy, but it probably also reflected the increasing will of patients to find alternatives to (effective or ineffective) antibiotics. It is therefore important to understand that desperate patients take the matter in their own hands and try to find alternative therapeutic options.

Only 15 (5.8%) of the 260 phage therapy requests resulted in actual phage therapy. Two hundred and forty five requests were rejected for diverse reasons (Figure 1):

- 70 applicants (26.9%) did not respond to the email request for more information;
- 124 requests (47.7%) concerned bacterial pathogens against which the QAMH had no potent phages available;
- 46 applications (17.7%) did not meet the other two eligibility criteria (antibiotic treatment failure and/or absence of other therapeutic options);
- 5 (25%) out of the 20 infecting bacterial strains for which a phagogram was performed were found to be non-susceptible to the available phages.

In most cases, the rejected applications were referred to reputable phage therapy centers abroad.

The high frequency of non-responsiveness of applicants to the initial information request could have been partly due to an inability to provide the necessary information. For instance, access to medical data could have been hampered by a lack of confidence from the treating physicians. This could have been partially remedied by conducting randomized controlled trials to demonstrate phage therapy efficacy and by awareness campaigns. The QAMH is developing tools, such as a comprehensive website with information and instructions, to dispense phage therapy information to health care professionals.

Almost half of the requests (47.7%) had to be dismissed because no suitable phages were available to treat the causative bacterial agents, which increasingly belong to the family of the *Enterobacteriaceae*. This observation prompted us to initiate research programs to isolate and characterize potent phages against, amongst others, problematic *E. coli* and *K. pneumoniae* strains. For this, we will need to address the remarkably high phage specificity within the *Enterobacteriaceae* family. Of importance, in this cohort 30 bacterial species were at the basis of the infections for which phage therapy was pursued. This observation highlights the main difficulty with which a phage therapy center is confronted. Indeed, to be able to cope with these 30 bacterial species, hundreds of potent and characterized phages need to be readily available or isolated de novo from the environment and produced to a quality acceptable for human application. As a consequence, big phage repositories will be required. This observation shows the importance of directing a considerable part of R&D efforts towards new technologies (e.g., synthetic biology) [17], which would allow the accelerated selection and production of potent therapeutic phages, for every possible pathogen.

Forty-six requests (17.7%) did not involve antibiotic treatment failure and/or the absence of other therapeutic options than phage therapy. Indeed, to be able to apply article §37 of the Declaration of Helsinki, a physician must be certain that “proven prophylactic, diagnostic and therapeutic methods do not exist or have been ineffective”. This is also a condition for phage therapy in the Wroclaw Ludwik Hirszfeld Institute. However, we found that the majority of cases did not involve MDR infection. If phage therapy requests had predominantly been made by (university) hospitals instead of individuals, the proportion of MDR cases would likely have been greater.

It must be said that some medical conditions such as COPD, bronchiectasis, and diabetic foot infection can be very difficult to treat due to underlying complications such as poor blood flow, a weakened immune system, or the presence of highly protected bacterial communities (in biofilms), even when the infecting bacterium is susceptible to common antibiotics. The difficulty is to demonstrate this unambiguously. The implementation of the magistral phage framework, which does not require the proven ineffectiveness of conventional therapies, should solve these issues in the near future in Belgium [9].

Currently, the limited (as compared to renown phage therapy centers, such as the Eliava Institute in Tbilisi) phage therapy expertise in the QAMH mainly concerns military- or mass casualty-associated indications such as burn wound and orthopedic infections, and to some extent respiratory diseases. Based on this analysis of phage therapy requests, we will expand our ability, capacity, and experience (including adequate treatment protocols) to treat other pathologies such as urological infection with phages.

Finally, we must keep in mind that phages are not “miracle drugs”, as antibiotics were once presented, but that they are probably only useful as additional tools in certain indications and conditions, which still need to be determined. As such, more phage therapy randomized controlled trials are needed, and phage antibiotic synergy (PAS) should be further explored [18,19].

With this report, we hope to help and guide the “phage therapy centers in the making”, which are slowly emerging from the “phage-averse” setting called Western medicine.

## Figures and Tables

**Table 1:** Demographics and microbiology of patients requesting phage therapy at the Queen Astrid military hospital (*n* = 260).

Infection Types		LRTI	UTI	SSTI	ENTI	BoneI	OPI	AbdI	ND	Other	Total
Demographics											
Number of requesters		59	79	21	22	16	14	9	12	28	260
Age	≤14	1	3	3	1				1		9
	15–29	2	1			1			1		5
	30–59	5	11	5	3	4	2	1	1	5	37
	≥60	21	26	9	6	4	10	2	1	2	81
	ND	30	38	4	12	7	2	6	8	21	128
Gender	Male	23	33	12	13	10	11	5	7	20	134
	Female	36	46	9	9	6	3	4	5	8	126
Countries	The Netherlands	38	69	5	14	5	7	5	10	21	174
	Belgium	9	4	12	2	5	6	3	2	7	50
	France	5	3	3	4	3	1				19
	Germany	3			2	1					6
	Luxembourg	1	2								3
	Italy			1							1
	Spain	1									1
	United States					2		1			3
	Israel	2									2
	Unknown		1								1
Bacterial pathogens											
*Pseudomonas aeruginosa*		36	7	8	9	3		2	1	4	70
*Escherichia coli*		3	29	2	3	2	2	2		1	44
*Staphylococcus aureus*		11		6	5	2	5	1	1		31
*Klebsiella pneumoniae*			18	1		2	1		2		24
*Enterococcus faecalis*			12		1					4	17
*Proteus mirabilis*		4	5		1	1					11
*Enterobacter cloacae*			6								6
*Mycobacterium avium*		3									3
*Streptococcus pyogenes*		1	2		1	1					5
*Staphylococcus epidermidis*						3	2		1		6
*Staphylococcus dysgalactiae*					1	2					3
*Acinetobacter baumannii*		2		1					1		4
*Serratia marcescens*		1		1							2
*Staphylococcus capitis*		1									1
*Staphylococcus warneri*							2				2
*Borrelia burgdorferi*										2	2
*Burkholderia cenocepacia*		1									1
*Burkholderia multivorans*		1									1
*Enterobacter aerogenes*				2							2
*Granulicatella adiacens*										2	2
*Haemophilus influenzae*		1									1
*Morganella morganii*						1				1	2
*Moraxella catarrhalis*		1									1
*Cutibacterium acnes*										2	2
*Stenotrophomonas maltophilia*		1									1
*Yersinia enterocolitica*									1		1
*Coxiella burnetii*										1	1
*Clostridium hathewayi*				1							1
*Helicobacter pylori*								1			1
*Corynebacterium amycolatum*					1						1
ND		8	22	3	5	2	2	3	5	12	62
Total		75	101	25	27	19	14	9	12	29	311
Polymicrobial (caused by a combination of bacteria)		10	14	4	4	3				2	37

AbdI, abdominal infection; BoneI, bone infection; ENTI, ear-nose-throat infection; LRTI, lower respiratory tract infection; ND, no data; OPI, orthopedic prosthesis infection; SSTI, skin and soft tissue infection; UTI, urinary tract infection.

## Supplementary Materials

The following are available online at https://www.mdpi.com/1999-4915/11/3/265/s1, Supplementary Table S1: Antibiograms reported for 102 infecting bacterial pathogens and Supplementary File 1: Phage therapy questionnaire.
