# Supplementary material for: Processing Phage Therapy Requests in a Brussels Military Hospital: Lessons Identified
Source: Viruses. 2019 Mar 17;11(3):265. doi: 10.3390/v11030265 (PMC6466067; doi:10.3390/v11030265)
Supplement: Supplementary file 1 [file viruses-11-00265-s001.zip › viruses-458048 revise supplementary/viruses-458048 revise supplementary file 1.docx]

**Demographic details of patient**: Date **Click here to enter a date.**

Name Type Here

SURNAME (Family Name) Type Here

Date of birth **Click here to enter a date.**

Age 00 year old

**Contact:**

Email address

Telephone number

Address

**Physician contact:**

Name

Specialty/GP

Email address

Phone number

Address

**[REQUIRED TO BE FILLED IN BY PHYSICIAN]**

| **Relevant past medical history (specify the date of diagnosis)**  **Medical**  **Surgery**  **Familial** |
| --- |

| **Current medication** |
| --- |

| **Allergies** |
| --- |

**DIAGNOSIS of INFECTION (incl. localization) [*Name Physician who filled form in*]**

*e.g. Right Shoulder Prosthetic Joint Infection/ CF with chronic colonisation of pseudomonas/..*

Date of diagnosis? ***Click here to enter a date.***

**Based on?**

**- Clinical features? (*include report of clinical features*) Yes**  **No**

*e.g. Fever, Pain*

**- Medical imaging? (*include report of medical imaging*) Yes  No**

*e.g. Rx, Mri*

**- Microbiology? (*include reports of microbiology*) Yes  No**

*e.g. Pseudomonas*

* A clear diagnosis based on clinical features, imaging and **microbiology** is important for eligibility of the case. If no specimen or taken in the past and if no antibiotic regimen was initiated, then eligibility is not possible

- Last AB-regimen + length of use? (for eligibility, all AB options need to be ruled out – Declaration of Helsinki)

*Type Here*

- Last surgery or debridement? (if applicable)

*Type Here*

- If the infection is still active, please specify near future planning (chronic AB?, which one? Suggested length of use?…)

*Type Here*

Please note that the last AB-gram is important for interpretation of multi-drug resistancy !!!

Please note that previously used or chronic use ABs are taken into account for eligibility

**Relevant Summary of current infectious disease HISTORY (in chronological order)**

| **Date (month-year)** | **Clinical features – Relevant lab results – Specimen of site of infection** | **Germs** | **AB use**  **(dose, frequency, route of admin, length of total use)**  **Date of initiation and stop** |
| --- | --- | --- | --- |
| *e.g.*  *Nov 2016* | *e.g. blood cultures, punction, …* | *e.g. pseudomonas aeruginosa* | *e.g. ciprofloxacin 750 mg 2dd PO during 6 weeks*  *start: / /*  *stop: / /* |
|  |  |  |  |
|  |  |  |  |

| Don’t forget to attach last blood analysis and last microbiology reports |
| --- |
